# Assessing the Dimensional Structure and Measurement Invariance of STABLE-2007 Scores in a California Sample of Adult Men on Parole for Sex Crimes

**DOI:** 10.5964/sotrap.19019

**Published:** 2026-09-08

**Authors:** Ryan E. Ditchfield, Allen Azizian, Deirdre D'Orazio, Lea Chankin

**Affiliations:** 1 Department of Criminology, California State University, Fresno, CA, USA; 2 Department of State Hospitals, SVP-Conditional Release Program, Sacramento, CA, USA; 3 California Department of Corrections and Rehabilitation – Division on Adult Parole Operations, Sacramento, CA, USA; Centre for Criminology (Kriminologische Zentralstelle – KrimZ), Wiesbaden, Germany; Medical Center - University of Freiburg, Freiburg, Germany

**Keywords:** STABLE-2007, sexual offending, risk assessment, construct validity, measurement invariance, exploratory structural equation modeling

## Abstract

The STABLE-2007 is a 13-item instrument used to identify, treat, and monitor changes in criminogenic needs associated with sexual reoffending, but no empirical research to date has examined the construct validity of this instrument within California. The present study assessed the factor structure and measurement invariance of STABLE-2007 scores in a Californian sample of 873 parolees enrolled in a community sex offense treatment program. Archival scores for the STABLE-2007 were compared across four time points over the course of supervision. Consistent with the broader literature on sexual offending risk, we found preliminary support for an invariant two-factor structure consisting of *antisociality* and *atypical sexuality*. In addition to providing support for the STABLE-2007’s construct validity and reliability, our identification of two clear constructs reflecting *antisociality* and *atypical sexuality* lays a foundation for establishing the instrument’s validity and reliability within the context of United States community correctional populations.

## Highlights

- This study examined the construct validity of the STABLE-2007 in a California sample.
- Two sexual offending risk constructs were identified: *antisociality* and *atypical sexuality.*
- These two constructs remained consistent over the course of community supervision.
- These findings extend the validity of this instrument to contexts beyond Canada and Austria.

Accurate interpretation of scores from psychological instruments, including risk assessment instruments like the STABLE-2007, hinges on the underlying validity and reliability of the assessment tool (Flake et al., 2017). Factor analysis is a class of statistical analyses used to establish validity in psychometric instruments (Brown & Moore, 2012; Strauss & Smith, 2009). Factor analysis is an indispensable analytic tool for assessing construct, convergent, and discriminant validity while adjusting for measurement error and method effects. To the authors’ knowledge, however, no research has yet assessed the factor structure of the STABLE-2007 within the United States. This means that, although the STABLE-2007 is a commonly required tool for assessment of sexual recidivism risk around the world, the instrument’s construct validity has yet to be formally verified in populations beyond Canada and Austria.

To address this gap, the present study assessed the factor structure and measurement invariance for the STABLE-2007 risk assessment instrument within a California sample of male parolees convicted of sexual offenses. The current study draws on modern structural equation modeling approaches to 1) determine the factor structure of the STABLE-2007 within a robust field sample, 2) evaluate the convergent and discriminant validity of identified factors by comparing dynamic and static risk scores, and 3) assess the reliability of the STABLE-2007's factor structure over time. In doing so, this study clarifies the latent dynamic risk factors underlying sexual offending within United States correctional populations, thereby facilitating accurate and effective community supervision and risk assessment of individuals convicted of sexual offenses.

## Latent Risk Factors Related to Sexual Offending

Broadly, risk assessment instruments can be categorized as assessing either static factors or dynamic factors that can be changed via intervention (i.e., criminogenic needs). Criminogenic needs, specifically, are enduring yet changeable offender characteristics that represent long-term vulnerabilities related to reoffending and that can theoretically improve in response to treatment (Eisenberg et al., 2019; Mann et al., 2010; Seto et al., 2023). Whereas instruments like the Static-99R evaluate relatively unchangeable static factors (e.g., age, criminal history) as a baseline for recidivism risk, the STABLE-2007 assesses criminogenic needs related to sexual recidivism (Brankley et al., 2021; Phenix et al., 2017). The STABLE-2007’s focus on criminogenic needs is, therefore, designed to address the *Need Principle* of the Risk-Need-Responsivity framework (Bonta & Andrews, 2023; Seto et al., 2023).

Prior theory and research suggest that static and dynamic risk factors for sexual offending can be characterized by at least two constructs representing sexual criminality and antisociality (Brouillette-Alarie et al., 2016). The underlying nature of these factors, however, is dependent on risk assessment instrument characteristics and the specific context of an assessed correctional population. Reliability and validity are properties of scores within a given sample, not general properties of an instrument that can be automatically generalized to new contexts (Cook & Beckman, 2006). This means that revalidation of risk assessment instruments is essential for an instrument’s use in new correctional populations, highlighting the strong need for re-assessment of the STABLE-2007’s psychometric properties. Identifying the underlying dimensions of risk assessment instruments also has utility with respect to treatment and assessment of recidivism risk, as prior research has shown that general and sexual offending risk factors differentially predict parolee’s risk for reoffending (Babchishin et al., 2016).

With regard to instruments assessing static risk, researchers have identified two to three static risk constructs. Brouillette-Alarie and Hanson (2015), for example, found a two-factor solution representing a rule-violation domain and a sex crime-specific domain, but a subsequent examination revealed a three-factor solution reflecting persistence/paraphilia, youthful stranger aggression, and general criminality (Brouillette-Alarie et al., 2016). By contrast, researchers have identified factor solutions ranging from two to four dimensions for dynamic risk assessment instruments. The Violence Risk Scale–Sexual Offending Version (VRS-SO), for example, has consistently produced scores reflecting a three-factor model consisting of sexual deviance, criminality, and treatment responsivity (Olver et al., 2018; Wielinga & Olver, 2024). The dimensionality of dynamic risk assessment instruments has not been fully resolved, however. Different solutions for the VRS-SO exist, including a four-factor solution in two samples of child sexual abusers (Beggs & Grace, 2011). More recent investigations of dynamic risk assessments’ construct validity have instead found support for a two-factor model consisting of sexual risk and antisocial opposition (e.g., Sex Offender Treatment Intervention and Progress Scale (SOTIPS); Miner et al., 2023).

## Prior Evidence Regarding the Dimensionality and Construct Validity of the STABLE-2007

The initial STABLE-2007 validation study by Hanson et al. (2007) did not specify a factor structure. Rather, STABLE-2007 items were initially selected by 1) empirically identifying supervision issues that predict sexual recidivism among individuals of similar static risk levels and 2) removing items that did not significantly predict sexual recidivism, such as sexual entitlement and attitudes tolerant of rape (Hanson & Harris, 1998, 2000). The STABLE-2007 coding manual, used as official training material in California, describes its 13 items as falling into five categories: Significant Social Relationships, Intimacy Deficits, Sexual Self-Regulation, General Self-Regulation, and Cooperation with Supervision (Fernandez et al., 2014, p. 14). The validity of this five-dimension model remains untested via peer-reviewed research, however.

Empirical research on the STABLE-2007’s construct validity, although sparse, has identified both two-factor and three-factor solutions for the STABLE-2007 (Brien-Robidoux, 2022; Brouillette-Alarie & Hanson, 2015; Etzler et al., 2020). Brouillette-Alarie and Hanson (2015) and Brien-Robidoux (2022) observed two constructs representing antisociality and sexual deviance, though the latter study also identified a third uninterpretable factor consisting of only Item 7 (Impulsive Acts). By contrast, Etzler and colleagues (2020) observed a three-factor solution representing antisociality, sexual deviance, and hypersexuality – with the caveat that the authors were forced to exclude Item 1 (Significant Social Influences) scores due to a lack of reliable information about offenders’ social status within the Austrian Prison System. Moreover, both Brien-Robidoux (2022) and Etzler et al. (2020) examined the STABLE-2007’s construct validity via exploratory factor analysis (EFA) but did not verify their identified constructs via confirmatory factor analysis (CFA) or exploratory structural equation modeling (ESEM).

Exploratory factor analysis, while an important initial step for assessing dimensionality, cannot provide the same level of information and clarity regarding factor structure as confirmatory factor analysis or modern equivalents such as exploratory structural equation modeling (Brown & Moore, 2012; Fabrigar et al., 1999; Flake et al., 2017). Confirmatory factor analysis, by contrast, is a theory-driven approach to assessing dimensionality that draws on structural equation modeling to allow testing of different model configurations and evaluation of model fit indices (Alamer, 2022; Woodrow, 2014). Unlike EFA, CFA can model the nature of relationships among items, account for measurement error, and allow for testing of hypothetical latent constructs and competing models. For these reasons, EFA is considered to be merely a first step in scale validation (Brown & Moore, 2012). To the authors’ knowledge, no research has yet investigated the dimensionality of the STABLE-2007 while applying best practices in factor analysis.

Other researchers have proposed alternative, hypothetical factor solutions for the STABLE-2007. Although they did not directly examine construct validity via exploratory or confirmatory factor analysis, Brankley et al. (2021, p. 51) argue that the STABLE-2007’s items assessing sexual recidivism risk may reflect several uniquely predictive aspects of sexual offending (e.g., risk for pedophilia vs. sexual sadism). This argument, notably, is consistent with the Structured Risk Assessment (SRA) Framework, a theoretical framework for dynamic risk assessment of men convicted of sexual offense (Thornton, 2002, 2013). The SRA framework includes four criminogenic needs domains: sexual interests, distorted attitudes, relational style, and self-management. In addition, each domain within the SRA framework is theoretically related to between two and three subdomains (e.g., the *self-management* domain consists of two sub-domains reflecting *social deviance* and *dysfunctional coping in response to stress/problems*). This framework, in other words, implies that the STABLE-2007 should assess at least four dimensions related to sexual recidivism. Yet, with the exception of recent network analyses applied to STABLE-2007 scores in Canada, Alaska, and Iowa, the interrelationships among these domains remain unexplored (Heffernan & Ward, 2017; van den Berg et al., 2020, 2022).

Beyond determining the dimensional structure of an instrument’s scores, demonstrating strong construct validity also requires assessment of convergent and discriminant validity (Strauss & Smith, 2009). Convergent validity refers to the degree that two measures that should be theoretically related are related. Conversely, discriminant validity refers to the degree that measures of constructs theoretically unrelated are in fact unrelated. Evidence regarding the STABLE-2007’s convergent and discriminant validity is limited and mixed. Smeth (2013) examined total score correlations between the STABLE-2007, Static-99R, and Acute-2007, determining that that the STABLE-2007 assesses different constructs than static (*r* = 0.28) and acute-dynamic (*r* = 0.30) risk. Nunes and Babchishin (2012) investigated the construct validity of STABLE -2007 by examining correlations between individual items and factors from other related scales (e.g., the RAPE and MOLEST scales and the UCLA Loneliness Scale, Bumby, 1996; Russell, 1996), concluding that STABLE items may be measuring different underlying constructs than those suggested by the original item names. Neither Smeth (2013) nor Nunes and Babchishin (2012), however, conducted formal analyses of the STABLE-2007’s factor structure. Instead, the authors relied on analysis of total scores or individual item scores, limiting the conclusions that could be drawn regarding the construct validity of latent risk factors within the STABLE-2007.

## Measurement Invariance: Assessing Reliability as a Prerequisite for Validity

Measurement invariance refers to the idea that the dimensional structure of a psychometric instrument should be consistent across time and contexts (e.g., cross-cultural comparisons; see Hagger et al., 2003; Megreya et al., 2016). Measurement invariance analysis, specifically, is an extension of confirmatory factor analysis that uses structural equation modeling to determine whether the factor structure of a psychometric instrument is stable across time and context (e.g., Megreya et al., 2016). Assessing invariance in the factor structure of psychometric instruments is a critical component of reliability in psychological assessment, as an unstable factor structure over time can be an indicator of low reliability in the instrument. Reliability is a necessary, yet insufficient, prerequisite for validity. Without consistency in measurement, one cannot conclude that an instrument is valid, even if a scale is shown to otherwise produce excellent predictive validity (Clifton, 2020; Cook & Beckman, 2006). By assessing measurement invariance over time, therefore, this study provides the necessary foundation for establishing that the STABLE-2007 is not only valid but reliable as a risk assessment instrument in the United States and elsewhere.

## Method

### Participants

All analyses were conducted using data from an existing database of 873 adult male parolees who received between two and four STABLE-2007 ratings while undergoing community supervision. The average time between the first and fourth STABLE-2007 assessment ranged from 1 to 4 years (*Mdn* = 3.00, *M* = 2.70, *SD* = 0.66). All participants were rated on the STABLE-2007 at least twice, 488 participants were rated on the STABLE-2007 at least three times, and 171 participants were rated at least four times. Given that fewer than 5% of participants received five or more STABLE-2007 ratings (*n* = 41, 4.70%), we restricted our analyses to only those participants who completed between two and four assessments. Demographic and descriptive statistics for our sample are provided in Table 1. This study was approved by the CDCR Research Oversight Committee, the California State University, Fresno Institutional Review Board (IRB), and the California Committee for the Protection of Human Subjects, which serves as the IRB for all California state agencies.

**Table 1:** Demographic and Descriptive Information (N = 873)

Variables	*M* (*SD*)	% (valid *n*)
Demographic information		
**Age at program entry**	44.03 (13.60)	99.5% (869)
Ethnicity		99.4% (868)
African American		21.6% (189)
Asian		2.4% (21)
American Indian		2.1% (18)
Hispanic		38.1% (333)
White		30.9% (270)
Other		4.2% (37)
Risk scales		
**STATIC-99R Total Score**	2.93 (2.62)	97.9% (855)
STABLE-2007 Total Score^a^	5.96 (3.79)	100.0% (873)
Time I	6.60 (4.44)	100.0% (873)
Time II	5.63 (4.15)	100.0% (873)
Time III	5.19 (3.78)	55.9% (488)
Time IV	5.37 (3.90)	19.6% (171)

*Note.* Subsample size (*n*) fluctuates as a function of missing data.

^a^Fewer than 5% of respondents provided scores on assessment time points V through VII (*n* = 41, 4.70%), so we restricted our factor analyses to assessment time points I through IV. The grand mean for STABLE-2007 scores (*M* = 5.96, *SD* = 3.79), however, was calculated based on all available data.

### Treatment Program Description

Sex offender treatment programs in California for individuals who are registered and under probation or parole supervision are required to meet standards established by the California Sex Offender Management Board (CASOMB). These standards are grounded in the Risk, Need, and Responsivity (RNR) principles that guide assessment and treatment of individuals convicted of sexual offenses. Assessment includes the Static-99R to establish baseline risk and inform the intensity of treatment, with the STABLE-2007 and Level of Service/Case Management Inventory (LS/CMI) administered at intake and annually to monitor changes in risk and treatment needs. Each agency has internal procedures for storing and using assessment data. In addition to standardized protocols for treatment completion, maintenance, and aftercare, agencies are also required to report assessment results, including individual item scores, through a government-maintained automated reentry management system. The data used in the present study were obtained from this government reporting system, which is commonly referred to as the Automated Reentry Management System (ARMS). Treatment is guided by a combination of internally developed and commercially available manuals, together with cognitive-behavioral and social learning approaches designed to address dynamic risk factors.

### Measures

#### STABLE-2007

The STABLE-2007 is a risk assessment instrument designed to assess 13 criminogenic needs among men convicted of sexual offenses (Hanson et al., 2007). It includes the following items: (1) Significant Social Influences, (2) Capacity for Relationship Stability, (3) Emotional Identification with Children, (4) Hostility Toward Women, (5) General Social Rejection/Loneliness, (6) Lack of Concern for Others, (7) Impulsive Acts, (8) Poor Cognitive Problem Solving, (9) Negative Emotionality, (10) Sex Drive and Preoccupation, (11) Sex as Coping, (12) Deviant Sexual Interests, and (13) Cooperation with Supervision. Each item is scored on a 3-point-rating scale coded as 0 for “no problem,” 1 for “some concern/slight problem,” and 2 for “present/definite concern.” Items are evaluated independently via a semi-structured interview, review of the individual’s criminal history, and by considering an individual’s prospective functioning presuming conditions of freedom. Assessed individuals are assigned a total score ranging between 0 and 26, with higher scores indicating a higher risk of recidivism. Meta-analytic reviews of previous research (Brankley et al., 2021) indicate that the Stable-2007 instrument produces scores with strong interrater reliability (median ICC = .90) and moderate predictive validity (AUC = .674, 95% CI [.646, .702]).

#### Static-99R

The Static-99R is an actuarial risk assessment instrument designed to assess 10 static risk factors among men convicted of sexual offenses (Helmus et al., 2022; Phenix et al., 2017). It includes the following items: (1) Age at release, (2) Any live-in intimate relationship for 2 or more years, (3) Convictions for index non-sexual violence, (4) Prior convictions for non-sexual violence, (5) Prior sex offenses, (6) Four or more prior sentencing dates, (7) Convictions for noncontact sexual offenses, (8) Any unrelated victims, (9) Any stranger victims, and (10) Any male victims. Items are evaluated via a review of the individual’s demographic and criminal history, with total scores ranging from –3 to 12. With the exception of Items 1 and 5, items are scored as either 0 (*not present*) or 1 (*present*). Item 1 is scored on a scale from –3 (*age 60 or older*) to 1 (*age 18 to 34.9*). Item 5 is scored on a scale from 0 (*no charges or convictions*) to 3 (*six or more charges or 4 or more convictions*). Only total scores were provided in this dataset. Similar to the STABLE-2007, meta-analytic reviews indicate that the Static-99R instrument produces scores with strong interrater reliability (ICCs ranging between .84 and .95) and moderate predictive validity (Helmus et al., 2022; fixed effect AUC = .678, 95% CI [.669, .668]; random effects AUC = .689, 95% CI [.671, .706]).

### Data Analysis

#### Exploratory Structural Equation Modeling

To assess the underlying dimensions of STABLE-2007 scores within our sample, we conducted exploratory structural equation modeling (ESEM) for item scores at each of the first four assessment time points (Asparouhov & Muthén, 2009; Marsh et al., 2014). Exploratory structural equation modeling, which applies an EFA measurement model with rotations within a structural equation modeling framework, integrates the rigor and parsimony of Confirmatory Factor Analysis (CFA) with the flexibility of Exploratory Factor Analysis (EFA) (for a detailed review of ESEM, see Marsh et al., 2014). The advantage of ESEM over CFA is that it allows for items to cross-load with other factors, whereas CFA forces cross-loadings to be 0. ESEM thereby addresses several issues with traditional CFA approaches, in that even small cross-loadings (<.100) can bias parameter estimates and it is extraordinarily rare for psychological constructs to be completely independent from one another (Asparouhov et al., 2015; Morin et al., 2016).

We constructed our ESEM models via the *psych* and *lavaan* packages in R (Revelle, 2025; Rosseel, 2012). Given the ordinal structure of STABLE-2007 items, we assumed a polychoric correlation structure and therefore extracted factors using mean-and-variance-adjusted (WLSMV) estimation (Goretzko et al., 2021; Holgado-Tello et al., 2010); extracted factors were rotated obliquely via the Oblimin method. The number of factors retained was based on parallel analysis (Horn, 1965), Velicer’s MAP criterion (Velicer, 1976; Velicer et al., 2000), and comparative model fit across the first four assessment time points.

We evaluated the fit of our final model based on the following indices: (a) the mean-and-variance-adjusted chi-square statistic (Asparouhov & Muthén, 2021), (b) the robust root mean square error of approximation (R-RMSEA) with a value smaller than or equal to .05 indicating a good fit and a value smaller than or equal to .08 indicating acceptable fit (Savalei, 2018; Schermelleh-Engel et al., 2003), (c) the standardized root mean residual (SRMR) with a value smaller than or equal to .08 indicating good fit (Hu & Bentler, 1999), and (d) the robust comparative fit index (R-CFI) with a value of .97 or higher indicating good fit and a value of .95 or higher indicating acceptable fit (Hu & Bentler, 1999; Savalei, 2018). Robust RMSEA and CFI values were used instead of traditional RMSEA and CFI values due to the STABLE-2007’s ordinal data structure, as robust indices are calculated using the mean-and-variance-adjusted chi-square statistic (Asparouhov & Muthén, 2021; Pavlov et al., 2020). Likewise, we report ordinal alpha coefficients (ordinal α) to assess each identified factor’s internal consistency via the *misty* package in R (Yanagida, 2025; Zumbo & Kroc, 2019; Zumbo et al., 2007).

#### Measurement Invariance

We assessed measurement invariance over time for the two-factor model using multiple-group confirmatory factor analysis (MG-CFA) via the *semTools* and *Lavaan* packages in R (Jorgenson et al., 2026; Rosseel, 2012). Given the ordinal structure of STABLE-2007, we followed guidelines for assessing invariance with categorical outcomes outlined by Wu and Estabrook (2016) and Svetina et al. (2020). The first and least restrictive step is to test a *baseline* or *configural* variance model wherein the number, pattern, and thresholds for parameters are assumed to be equal across time points, but values of parameters are free to vary. The second step is to test a *metric* invariance model wherein the pattern and values of factor loadings are assumed to be equivalent across time points. The final step is to test a *scalar* invariance model wherein both loadings and intercepts are assumed to be equal across time points. We used Svetina and Rutkowski’s (2017) recommendations regarding measurement invariance cutoffs for ordinal data: (1) ΔRMSEA ≤ .05 in conjunction with significant Δχ^2^ as evidence of *metric* invariance and (2) ΔRMSEA ≤ .01 and ΔCFI ≤ −.002 as evidence of *scalar* invariance.

## Results

### Exploratory Structural Equation Modeling

Parallel analysis and Velicer’s MAP test suggested viable factor solutions ranging from 1 to 5 across all assessment time points. To select a final model for assessment of measurement invariance, we estimated and compared model fit for one-, two-, three-, four-, and five-factor solutions at each time point, the results of which are presented in Table 2. Although a four-factor model technically produced the strongest model fit statistics, both the two-factor (R-RMSEA ≤ .049, SRMR ≤ .070, CFI ≥ .963) and three-factor (R-RMSEA ≤ .040, SRMR ≤ .053, CFI ≥ .983) models met criteria for strong model fit across all time points. Therefore, following the principle of parsimony (Falk & Muthukrishna, 2023), we selected the two-factor solution as our final model for the purpose of measurement invariance evaluation. To aid future replication efforts, we provide a detailed overview of the two-, three-, and four-factor ESEM solutions below.

**Table 2:** Comparisons of Exploratory Structural Equation Model (ESEM) Fit Across Assessment Time Points

Model	χ^2^ (*df*)	R-RMSEA	SRMR	R-CFI
Time I (*n* = 873)				
1-factor model	366.23 (65)	.060	.069	0.928
2-factor model	249.51 (53)	.049	.050	0.966
3-factor model	159.27 (42)	.040	.039	0.986
4-factor model	107.16 (32)	.035	.030	0.994
5-factor model^a^	272.57 (33)	.079	.058	0.808
Time II (*n* = 873)				
1-factor model	285.38 (65)	.054	.067	0.952
2-factor model	189.76 (53)	.041	.044	0.982
3-factor model	114.21 (42)	.031	.032	0.996
4-factor model	64.36 (32)	.023	.022	1.000
5-factor model	35.38 (23)	.016	.015	1.000
Time III (*n* = 488)				
1-factor model	159.01 (65)	.048	.058	0.945
2-factor model	123.86 (53)	.043	.046	0.964
3-factor model	68.80 (42)	.028	.033	0.988
4-factor model	35.20 (32)	.011	.021	0.999
5-factor model	20.19 (23)	.000	.015	1.000
Time IV (*n* = 171)				
1-factor model	93.54 (65)	.049	.082	0.941
2-factor model	73.51 (53)	.043	.070	0.963
3-factor model	52.76 (42)	.032	.053	0.983
4-factor model	31.91 (32)	.000	.032	1.000
5-factor model*^a^*	160.84 (33)	.149	.120	0.721
All Times (*n* = 2,405)				
1-factor model	781.58 (65)	.055	.064	.938
2-factor model	524.69 (53)	.044	.044	.969
3-factor model	307.81 (42)	.035	.035	.986
4-factor model	159.38 (32)	.027	.027	.995
5-factor model	76.96 (23)	.020	.014	.999

*Note.* χ^2^(*df*) = mean-and-variance corrected chi-square (degrees of freedom). R-RMSEA = robust root mean square error of approximation. SRMR = standardized root mean residual. R-CFI = Robust Comparative Fit Index.

^a^Model could not be identified when assuming correlated (oblique) factors; re-estimated assuming independent (orthogonal) factors.

**Figure 1:**
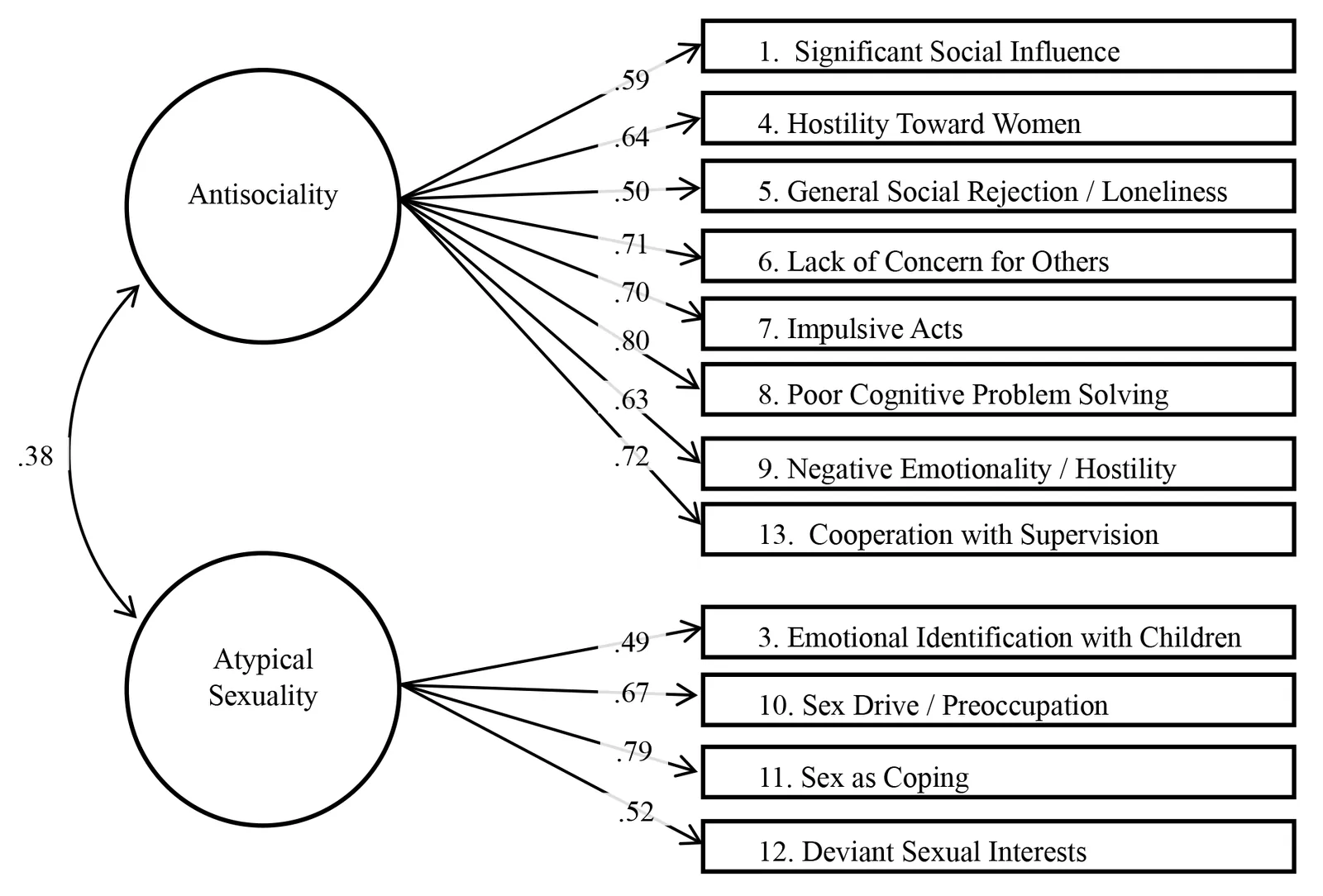
ESEM Analysis of STABLE-2007 Item Scores for All Time Points: Correlated Two-Factor Model *Note.* ESEM = Exploratory Structural Equation Modeling. Item 2 (“Capacity for Relationship Stability”) did not load onto either factor (λ*_Antisociality_* = 0.26; λ*_AtypicalSexuality_*= 0.18).

The two-factor solution, as shown in Figure 1 below, resulted in two highly interpretable constructs (|λ|*_Antisociality_* = 0.502−0.796, *M* = 0.661, ordinal α = 0.86; |λ|*_AtypicalSexuality_* = 0.491−0.795, *M* = 0.618, ordinal α = 0.66). The first factor consisted of eight items reflecting *antisociality*: (1) Significant Social Influences, (4) Hostility Toward Women, (5) General Social Rejection, (6) Lack of Concern for Others, (7) Impulsive Acts, (8) Poor Cognitive Problem Solving, (9) Negative Emotionality, and (13) Cooperation with Supervision. The second factor consisted of four items reflecting *atypical sexuality*: (3) Emotional Identification with Children, (10) Sex Drive and Preoccupation, (11) Sex as Coping, and (12) Deviant Sexual Interests. Item 2 (Capacity for Relationship Stability), however, did not load onto either factor (λ*_Antisociality_* = 0.264; λ*_AtypicalSexuality_* = 0.182). Our two-factor solution suggests that California adult male parolees’ scores on the STABLE-2007 may reflect two sources of risk: antisocial tendencies and atypical sexuality.

The three-factor solution, similarly, resulted in *antisociality* and *atypical sexuality* constructs (|λ|*_Antisociality_* = 0.478−0.734, *M* = 0.649, ordinal α = 0.86; |λ|*_AtypicalSexuality_* = 0.432−0.762, *M* = 0.646, ordinal α = 0.65), but also identified a third *asocial relations* construct consisting of two items: general loneliness and emotional identification with children (|λ|*_AsocialRelations_* = 0.453−0.594, *M* = 0.524, ordinal α = 0.46). The four-factor solution, in addition to *atypical sexuality* (|λ|*_AsocialRelations_* = 0.421−0.774, *M* = 0.640, ordinal α = 0.78), split the *antisociality* construct into three smaller constructs representing *asocial relations* (|λ|*_AsocialRelations_* = 0.469−0.561, *M* = 0.515, ordinal α = 0.46), *negative emotionality* (|λ|*_NegativeEmotionality_* = 0.630−0.793, *M* = 0.716, ordinal α = 0.80), and *impulsive antisociality* (|λ|*_ImpulsiveAntisociality_* = 0.491−0.770, *M* = 0.644, ordinal α = 0.65). Item 2 (Capacity for Relationship Stability), once again, did not load onto any factor across the three- or four-factor solutions.

### Convergent and Discriminant Validity: Changeable Versus Static Risk

Next, we assessed for convergent and discriminant validity between our identified *antisociality* and *atypical sexuality* changeable risk factors and static risk factors by calculating Spearman’s rho correlations among STABLE-2007 scores combined across all four assessment time points and initial Static-99R scores. Antisociality scores exhibited weak convergence with static risk (*r_s_* = .375, *p* < .001) and atypical sexuality scores (*r_s_* = .238, *p* < .001). Atypical sexuality scores exhibited strong discriminant validity when compared to static risk scores (*r_s_* = −.028, *p* = .416). But, whereas the *antisociality* factor exhibited good internal consistency (ordinal α = 0.86), the *atypical sexuality* factor exhibited relatively weak internal reliability (ordinal α = 0.66), suggesting that the items within the *atypical sexuality* factor might be assessing multiple, modestly related, yet distinct aspects of atypical sexuality.

### Measurement Invariance

For conciseness, we provide a summary and comparison of model fit indices across measurement invariance models in Table 3. Overall, we observed evidence for measurement invariance across time in our sample. Specifically, scores passed the tests for both metric invariance (ΔRMSEA = −.003; Δχ^2^_(30)_ = 49.92, *p* = .013) and scalar invariance (ΔRMSEA = −.003; ΔR-CFI = .000).

**Table 3:** Summary of Measurement Invariance Analyses for Two-Factor Model

Model	χ^2^ (*df*)	R-RMSEA	SRMR	R-CFI
Model fit indices				
Configural model	765.37 (212)***	.066	.081	0.934
Metric model	815.29 (242)***	.063	.084	0.932
Scalar model	843.17 (272)***	.059	.088	0.932
Differences in model fit				
Configural vs. Metric	49.92 (30)*	−.003	.003	−0.002
Metric vs. Scalar	27.88 (30)	−.004	.004	0.000

*Note.* All models estimated using the two-factor solution identified via exploratory structural equation modeling. χ^2^ (*df*) *= mean-and-variance corrected chi-square (degrees of freedom).* R-RMSEA = robust root mean square error of approximation. SRMR = standardized root mean residual. R-CFI = Robust Comparative Fit Index.

*p < .05. **p < .01. ***p < .001.

## Discussion

Using a modern structural equation modeling approach, the present study examined the dimensional structure and measurement invariance properties of STABLE-2007 scores in a sample of male parolees who were mandated to participate in sexual offense treatment. Consistent with prior research on risk assessment for sexual offenses (Brouillette-Alarie & Hanson, 2015; Brouillette-Alarie et al., 2016), we identified a time-invariant two-factor solution. The first factor reflected an *antisociality* construct and the second factor reflected a *atypical sexuality* construct. These *antisociality* and *atypical sexuality* constructs were conceptually distinct from each other and from static risk measured via Static-99R scores. Multiple-group confirmatory factor analysis confirmed that this two-factor structure exceeded empirical standards for both metric invariance (ΔRMSEA = −.003) and scalar invariance (ΔRMSEA = −.003; ΔR-CFI = .000), indicating that the *antisociality* and *atypical sexuality* constructs exhibit reliability with respect to dimensional structure over time (Svetina & Rutkowski, 2017). Notably, our two-, three, and four-factor solutions do not align with the informal five-factor model currently used in official STABLE-2007 training materials in California (Fernandez et al., 2014). Our findings suggest that these training materials and the associated coding manual may need to be updated to reflect a more accurate representation of the changeable risk factors measured by the STABLE-2007.

### Evaluating the Dimensionality of the STABLE-2007

By using a modern ESEM approach to assess factor structure and measurement invariance, our study provides the most statistically sophisticated examination of STABLE-2007’s dimensionality to date. Whereas prior research has assessed the STABLE-2007’s dimensionality via exploratory factor analysis or network analysis of individual items (Brouillette-Alarie & Hanson, 2015; Etzler et al., 2020; van den Berg et al., 2020, 2022), exploratory structural equation modeling (ESEM) enabled us to clarify the instrument’s factor structure by integrating the rigor of Confirmatory Factor Analysis (CFA) with the flexibility of Exploratory Factor Analysis (Asparouhov & Muthén, 2009; Marsh et al., 2014). In doing so, our research establishes the STABLE-2007’s validity and reliability in California and broader United States correctional contexts.

Although we selected a two-factor solution as our final model, it is worth noting that the one-, three-, and four-factor models exhibited good or acceptable model fit across the four assessment time points included within our study. Even the one-factor solution, which had the “worst” fit to the scores within our sample, would still be considered acceptable by our model selection standards, even if it did not exceed all model selection criteria. We selected the two-factor model as our final model because it exceeded criteria for “good” or “excellent” fit at every time point while being more parsimonious than the three- and four-factor models (Falk & Muthukrishna, 2023). Our two-factor solution also aligned more strongly with past theoretical (Brouillette-Alarie et al., 2016; Olver et al., 2007; McGrath & Thompson, 2012) and empirical (Brien-Robidoux, 2022; Etzler et al., 2020; Hanson et al., 2007) solutions than the three- and four-factor solutions. We note, however, that the distinction between our two-factor solution within California correctional populations (*antisociality* & *atypical sexuality*) and the three-factor solution within Austrian correctional populations identified by Etzler and colleagues (2020; *antisociality*, *sexual deviance,* & *hypersexuality*) suggests a need for further cross-cultural validation of the STABLE-2007 instrument. That is, future research should investigate whether the dimensional structure of STABLE-2007 is susceptible to change across cultural contexts.

Our findings also underline the complexity of dynamic risk assessment constructs related to sexual offending. Whereas our first *antisociality* factor exhibited good internal consistency (ordinal α = 0.86), our second *atypical sexuality* factor exhibited moderately weak internal consistency (ordinal α = 0.66). The relatively low reliability of the *atypical sexuality* construct suggests that the items within Factor 2 may be assessing multiple, moderately related, yet distinct aspects of atypical sexuality. Notably, this finding is consistent with Brankley et al.’s (2021) argument that the STABLE-2007 items may be assessing several underlying dimensions of sexual criminality. Moreover, the low internal consistency for the *atypical sexuality* construct is consistent with broader theoretical frameworks for dynamic risk assessment of men convicted of sexual offense, most notably the Structured Risk Assessment (SRA) Framework (Thornton, 2002, 2013). The idea that atypical sexuality can be split into multiple sub-domains also aligns with recent, innovative approaches to measuring dimensionality via structured network analysis (van den Berg et al., 2020, 2022). Van den Berg et al.’s (2020) analysis of connections within the sexual-regulation-cluster, for example, mirrors Etzler et al.’s (2020) finding that *sexual preoccupation* and *sex as coping* form a factor conceptually separate from *deviant sexual interests*. We note, also, that the strong centrality of *general rejection/loneliness*, *poor cognitive problem solving*, and *impulsive acts* in van den Berg et al.’s (2020) data align with the high internal consistency for the *antisociality* factor observed in our sample scores.

Further supporting the idea that items related to relationships may be a unique construct within the STABLE-2007, the item *capacity for relationship stability* did not load onto any of the viable factor solutions identified within in our sample – even after combining the sample across all assessment time points to maximize statistical power. One possible explanation for this unexpected item loading pattern is that the *capacity for relationship stability* item was measured in a substantively different manner than the other items on the STABLE-2007 or reflects a distinct underlying construct. This is not to suggest, however, that the *capacity for relationship stability* item should be removed from the STABLE-2007 instrument. Instead, we highlight this item loading pattern to provide guidance on future STABLE-2007 research investigating the instrument’s predictive validity. In particular, researchers might consider testing whether *capacity for relationship stability* uniquely predicts recidivism risk above and beyond the *antisociality* and *atypical sexuality* constructs identified here. Prior research has already shown that STABLE-2007 items vary with respect to their potential for predicting reoffending. In van den Berg et al.’s (2020) shortest paths analysis, for example, *impulsive acts* shared the strongest independent relationship with sexual and violent recidivism, whereas *emotional identification with children* was directly related to sexual, but not violent, recidivism. In the event that *capacity for relationship stability* lacks unique predictive validity, researchers might next consider testing whether including or excluding this item from models improves the predictive validity of STABLE-2007 scores. Alternatively, in the event that this item retains unique predictive validity, researchers might consider creating and testing additional items that align with the underlying construct measured by the *capacity for relationship stability* item.

Our results also suggest that the *antisociality* construct could be represented by two or more specific subdomains. Specifically, our four-factor solution, consistent with recent research on Dark Tetrad personality traits and atypical sexuality, suggests that the broader *antisociality* domain could be feasibly split into two dimensions representing *negative emotionality* and *impulsive antisociality* (Costa et al., 2023; Lassche et al., 2024). Our three- and four-factor ESEM solutions also provide moderate evidence that general loneliness and emotional identification with children can be treated as an independent *asocial relations* construct that is distinct from both *atypical sexuality* and *antisociality* – though the reliability of this construct was low (ordinal α = 0.46). This finding aligns with prior research indicating that individuals with pedophilia tend to report higher levels of loneliness and social anxiety (Marshall et al., 2012; Schulz et al., 2017). We recommend that future research investigate whether these more complex solutions provide incremental predictive validity above and beyond our two-factor solution, as the ability of unique constructs to predict recidivism is highly useful in risk assessment (Mann et al., 2010).

Finally, we found strong support for the STABLE-2007’s ability to reliably measure our identified *antisociality* and *atypical sexuality* constructs over the course of community supervision. Specifically, scalar invariance in the dimensional structure of STABLE-2007 scores over time suggests that, within California, the two-factor solution for the STABLE-2007 benefits from clear structure and long-term construct validity, as previously observed in other contexts (i.e., Etzler et al., 2020). We emphasize, however, that these findings should be interpreted within the context of California parolees. Reliability and validity are properties of scores within a given sample, not properties of an instrument (Cook & Beckman, 2006). In other words, the validity and reliability characteristics identified within this study may not extend to correctional and treatment contexts beyond California specifically and the United States more generally. With this caveat in mind, the present findings provide strong preliminary evidence that the *antisociality* and *atypical sexuality* constructs within the STABLE-2007 can be measured reliably over time.

### Future Directions and Limitations

The present findings have several implications for treatment and evaluation of men convicted of sex offenses. Consistent with Smeth (2013), we observed moderate evidence for discriminant validity between the STABLE-2007’s *antisociality* and *atypical sexuality* dynamic risk constructs and static risk as measured by Static-99R total scores. These findings suggest that the antisociality and atypical sexuality factors identified in this study are distinct from one another and from static risk factors assessed by instruments such as the Static-99R. We could not, however, examine discriminant validity by comparing against Static-99R subscales in this study, as the available data did not include Static-99R item scores; we therefore recommend that future research examine the discriminant validity of instrument subscale scores, if possible.

Moreover, future research will need to verify the construct validity of our identified *antisociality* and *atypical sexuality* constructs by evaluating convergence with other scales assessing these constructs. Nunes and Babchishin (2012) previously observed low correlations between individual STABLE-2007 items and purportedly related scales such as the RAPE and MOLEST scales and the UCLA Loneliness Scale (Bumby, 1996; Russell, 1996). Assessing convergence using *antisociality* and *atypical sexuality* subscale scores rather than individual item scores might provide a clearer picture of the instrument’s convergent and discriminant validity with respect to relevant constructs such as impulsivity, loneliness, empathy, and sexual preoccupation. However, we acknowledge that restrictions on data collection within California correctional facilities may hamper or prevent administration of non-state-mandated assessment tools. Therefore, although imperfect, we recommend first assessing convergence with general criminality via other state-mandated instruments such as the LS\CMI (Jimenez et al., 2018; Schmidt et al., 2017).

Next, we highlight an important limitation for our approach to measurement invariance in this paper, namely that Svetina et al.’s (2020) multiple-group CFA framework cannot account for the assumption of dependence inherent to longitudinal designs. Although longitudinal measurement invariance models exist to address this issue (e.g., Liu et al., 2017; Mackinnon et al., 2022), these models were unsatisfactory solutions for the current data for several reasons. First, Liu et al.’s (2017) framework, while adequate at handling ordinal data structures, was developed for assessing unidimensional scales, not multidimensional scales like the STABLE-2007. Second, traditional longitudinal measurement invariance frameworks like the one developed Mackinnon et al. (2022) cannot account for the ordinal structure of the STABLE-2007, resulting in ill-fitting or unidentified models. At this time, Svetina et al.’s (2020) approach is the only measurement invariance approach, to the authors’ knowledge, that can both handle multidimensional scales and account for ordinal data structures. Given that the core finding of this paper is that the STABLE-2007 is best captured by two or more factors and that capturing these dimensions necessitates accounting for the instrument’s ordinal data structure, we determined that Svetina et al.’s (2020) multiple-group CFA framework offered a decent, albeit imperfect, compromise for assessing the reliability of the STABLE-2007’s factor structure over time. However, we highlight this issue as a critical area for future re-analysis once longitudinal measurement invariance models that account for multidimensional ordinal data are developed.

Finally, and perhaps most importantly, validation of the STABLE-2007 lays the foundation for future research examining the predictive validity of STABLE-2007 scores. There is still a strong need for additional evaluation of construct and predictive validity in risk assessment instruments, as several risk assessment instruments commonly used in California have been shown to have poor or moderate predictive validity (Fazel et al., 2022). The STABLE-2007 has previously been found to have moderate accuracy in predicting sexual recidivism (e.g., Brankley et al., 2021). Assessing the predictive validity of construct-specific scores rather than relying on the raw STABLE-2007 total scores, however, may improve the STABLE-2007’s ability to reliably predict general and sexual reoffending.

A construct-focused approach would also enable testing of whether *antisociality* or *atypical sexuality* is a stronger predictor of recidivism risk. Prior research on static risk suggests that the inclusion of sex-crime-specific items can undermine an instrument’s ability to predict general and violent recidivism (Babchishin et al., 2016). As noted by Brankley et al. (2021), this suggests that the STABLE-2007 *atypical sexuality* construct may be unrelated, or even negatively related, to nonsexual reoffending. If this hypothesis is supported, practitioners using the STABLE-2007 who are interested in predicting general or violent recidivism might benefit from stronger predictive validity if they rely on *antisociality* subscale scores rather than total scores.

### Conclusion

We observed moderate initial support for the validity and reliability of the STABLE-2007 risk assessment instrument within a sample of California parolees under community supervision. Consistent with prior theory regarding assessment of sexual recidivism risk, we identified two distinct time-invariant constructs in STABLE-2007 scores: *antisociality* and *atypical sexuality*. We recommend that future research build upon the established predictive validity of STABLE-2007 scores by examining the relative diagnostic utility of the *antisociality* and *atypical sexuality* constructs identified in this article within other United States correctional populations. We also recommend that training materials be updated to reflect the empirically verified constructs identified in this research, as accurate representation of risk factors may improve treatment and evaluation of men convicted of sex offenses.

## Supplementary Materials

Although we focused our results and discussion on the 2-factor ESEM model in this paper, we recognize that researchers may be interested in alternative models for the purpose of future replication and validation efforts. Accordingly, we have provided a supplementary table containing the factor loadings for the unselected 1-, 3-, and 4-factor ESEM models via the open-access repository PsychArchives (for access, see Ditchfield et al., 2026S).



DitchfieldR. E.
AzizianA.
D'OrazioD.
ChankinL.
 (2026S). Supplementary materials to "Assessing the dimensional structure and measurement invariance of STABLE-2007 scores in a California sample of adult men on parole for sex crimes"
[Supplementary table]. PsychOpen. 10.23668/psycharchives.22440
PMC1361014742787840

## Data Availability

The dataset used in this study contains potentially identifying and sensitive information and is not publicly available due to data privacy laws. Access to these data can be requested by contacting the California Department of Corrections and Rehabilitation (CDCR) Office of Research (email: data.requests@cdcr.ca.gov).
